# 1-Benzoyl-3-[4-(3-benzoyl­thio­ureido)phen­yl]thio­urea

**DOI:** 10.1107/S160053681004599X

**Published:** 2010-11-13

**Authors:** Wong Woei Hung, Mohammad B. Kassim

**Affiliations:** a School of Chemical Sciences and Food Technology, Faculty of Science and Technology, Universiti Kebangsaan Malaysia, UKM 43600 Bangi Selangor, Malaysia

## Abstract

The mol­ecule of the title compound, C_22_H_18_N_4_O_2_S_2_, lies across a crystallographic inversion centre. The mol­ecule adopts a *syn–anti* configuration with respect to the positions of the carbonyl groups and terminal phenyl rings relative to the thione S atom across the C—N bond. There are two intra­molecular N—H⋯O and C—H⋯S hydrogen bonds within each molecule, resulting in the formation of four six-membered *S*(6) rings. The central and terminal rings make a dihedral angle of 13.55 (15)°. In the crystal, mol­ecules are linked by inter­molecular C—H⋯S hydrogen bonds, forming *R*_2_^2^(14) rings and resulting in zigzag chains.

## Related literature

For related compounds and structural parameters, see: Hung *et al.* (2010 ▶), Thiam *et al.* (2008 ▶); Arslan *et al.* (2004 ▶); Yamin *et al.*, (2003 ▶). For bond-length data, see: Allen *et al.* (197) ▶. For hydrogen-bond motifs, see: Etter *et al.* (1990 ▶); Bernstein *et al.* (1995 ▶).
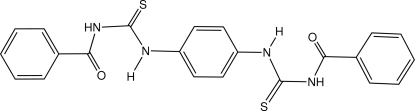


## Experimental

### 

#### Crystal data

C_22_H_18_N_4_O_2_S_2_
*M* *_r_* = 434.52
Monoclinic, 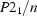

*a* = 11.513 (4) Å
*b* = 4.5279 (16) Å
*c* = 20.209 (7) Å
β = 101.146 (7)°
*V* = 1033.6 (6) Å^3^
*Z* = 2
Mo *K*α radiation
μ = 0.29 mm^−1^
*T* = 298 K
0.50 × 0.15 × 0.13 mm

#### Data collection

Bruker SMART APEX CCD area-detector diffractometer
Absorption correction: multi-scan (*SADABS*; Bruker, 2000 ▶) *T*_min_ = 0.950, *T*_max_ = 0.964
6173 measured reflections
2142 independent reflections
1529 reflections with *I* > 2σ(*I*)
*R*_int_ = 0.040

#### Refinement

*R*[*F* ^2^ > 2σ(*F* ^2^)] = 0.063
*wR*(*F* ^2^) = 0.171
*S* = 1.14
2142 reflections
136 parameters
H-atom parameters constrained
Δρ_max_ = 0.34 e Å^−3^
Δρ_min_ = −0.23 e Å^−3^

### 

Data collection: *SMART* (Bruker, 2000 ▶); cell refinement: *SAINT* (Bruker, 2000 ▶); data reduction: *SAINT*; program(s) used to solve structure: *SHELXS97* (Sheldrick, 2008 ▶); program(s) used to refine structure: *SHELXL97* (Sheldrick, 2008 ▶); molecular graphics: *ORTEPIII* (Burnett & Johnson, 1996 ▶), *ORTEP-3 for Windows* (Farrugia, 1997 ▶) and *PLATON* (Spek, 2009 ▶); software used to prepare material for publication: *SHELXTL* (Sheldrick, 2008 ▶) and *PLATON*.

## Supplementary Material

Crystal structure: contains datablocks I, global. DOI: 10.1107/S160053681004599X/dn2618sup1.cif

Structure factors: contains datablocks I. DOI: 10.1107/S160053681004599X/dn2618Isup2.hkl

Additional supplementary materials:  crystallographic information; 3D view; checkCIF report

## Figures and Tables

**Table 1:** Hydrogen-bond geometry (Å, °)

*D*—H⋯*A*	*D*—H	H⋯*A*	*D*⋯*A*	*D*—H⋯*A*
N2—H2*A*⋯O1	0.86	1.85	2.590 (3)	144
C11—H11⋯S1	0.93	2.56	3.215 (3)	128
C5—H5⋯S1^i^	0.93	2.84	3.567 (3)	136

Symmetry code: (i) 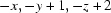.

**Fig. 1.**
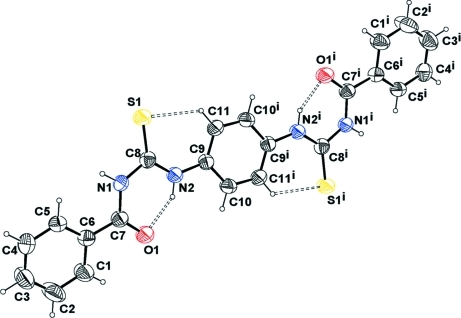
The molecular structure of the title compound with the atom labeling scheme. Displacement ellipsoids are drawn at the 50% probability level. H atoms are represented as small spheres of arbitrary radii. H bonds are shown as dashed lines.[Symmetry code: (i) -x+1, -y+2, -z+2.]

**Fig. 2.**
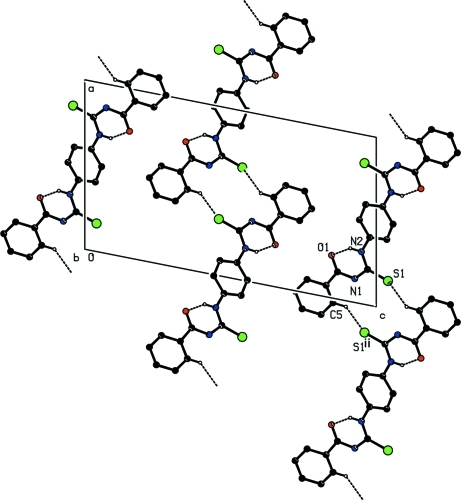
Partial packing view down the b axis showing the formation of R22(14) graph set motifs. Hydrogen bonds are drawn as dashed lines. H atoms not involved in hydrogen bondings have been omitted for clarity. [Symmetry code: (ii) -x, -y+1, -z+2]

## supplementary crystallographic information

### Comment

The title compound (Fig. 1) is a benzoyl thiourea derivatives and analogous to 1,2-bis(*N*'-benzoylthioureido)benzene, (Thiam *et al.*, 2008), except that the other thiourea moiety is located in *para* position of the centre benzene ring. It is also an isomer of 1,1'-Diphenyl-3,3'-(*p*-phenylenedicarbonyl)dithiourea which was reported perviously (Hung *et al.*, 2010). The bond lengths and angles are in normal ranges (Allen *et al.*, 1987). The C=O bond length of 1.227 (3)Å is longer than the average C=O bond length (1.200 Å) and comparable to that observed in *N*-benzoyl-*N*'-phenylthiourea (Yamin *et al.*, 2003). The C—N bond lengths are in the range of 1.330 (3) Å-1.415 (3)Å which are shorter than the normal single C—N bond length (1.469 Å) indicating double bond character (Arslan *et al.* 2004) owing to the resonance effect at the carbonyl-thiourea moiety. The thiourea fragment (S1/O1/N1/C6/C7/C8) is planar with a maximum deviation from its mean plane of 0.044 (3)Å for C8 atom. The central and terminal phenyl rings are essentially planar. The two rings make dihedral angles of 2.19 (13)° and 12.24 (15)°, respectively, with the thiourea fragment and the dihedral angle between those two rings is 13.55 (15)°.

As in most of the benzoyl thiourea derivatives, N–H···O intramolecular hydrogen bonding lead to the formation of two six membered S(6) rings [Etter *et al.*, 1990; Bernstein *et al.*, 1995) namely, C7/N1/C8/N2/H2/O1 and C7^i^/N1^i^/C8^i^/N2^i^/H2A^i^/O1^i^ (Fig. 1, Table 1). There are also weak C-H···S intramolecular hydrogen bonds involving resulting in another two S(6) rings (C8/N2/C9/C11/H11/S1 and C8^i^/N2^i^/C9^i^/C11^i^/H11^i^/S1^i^). In the crystal structure, molecules are linked by intermolecular C–H···S hydrogen bonds (Table 1) building *R*_2_^2^(14) rings (Etter *et al.*, 1990; Bernstein *et al.*, 1995) (Fig. 2). Owing to the fact that the molecule is organised around an inversion center, these rings extend on each side of the molecule to form azigzag chain.

### Experimental

The title compound was synthesized according to previously reported method with some modification (Thiam *et al.* 2008). Benzoyl chloride (10 mmol) was added to ammonium thiocyanate solution (10 mmol) and the mixture was left to react to completion. A yellowish product was filtered and added to a 1,4-diaminobenzene (5 mmol) in acetone and left at a refluxing temperature for 5 h. Yellowish precipitate was formed and a slow evaporation of the DMF solution of the product gave a crystal suitable for X-ray diffraction (Yield:75%).

### Refinement

All H atoms attached to C and N were calculated and treated as riding on their parent atoms with C-H= 0.93Å and N-H= 0.86Å with *U*_iso_=1.2*U*_eq_ (C, N).

### Crystal data

**Table:**

C_22_H_18_N_4_O_2_S_2_	*F*(000) = 452
*M**_r_* = 434.52	*D*_x_ = 1.396 Mg m^−^^3^
Monoclinic, *P*2_1_/*n*	Melting point: 511 K
Hall symbol: -P 2yn	Mo *K*α radiation, λ = 0.71073 Å
*a* = 11.513 (4) Å	Cell parameters from 1244 reflections
*b* = 4.5279 (16) Å	θ = 1.9–26.5°
*c* = 20.209 (7) Å	µ = 0.28 mm^−^^1^
β = 101.146 (7)°	*T* = 298 K
*V* = 1033.6 (6) Å^3^	Needle, yellow
*Z* = 2	0.50 × 0.15 × 0.13 mm

### Data collection

**Table:**

Bruker SMART APEX CCD area-detector diffractometer	2142 independent reflections
Radiation source: fine-focus sealed tube	1529 reflections with *I* > 2σ(*I*)
graphite	*R*_int_ = 0.040
ω scan	θ_max_ = 26.5°, θ_min_ = 1.9°
Absorption correction: multi-scan (*SADABS*; Bruker, 2000)	*h* = −11→14
*T*_min_ = 0.950, *T*_max_ = 0.964	*k* = −5→5
6173 measured reflections	*l* = −25→23

### Refinement

**Table:**

Refinement on *F*^2^	Primary atom site location: structure-invariant direct methods
Least-squares matrix: full	Secondary atom site location: difference Fourier map
*R*[*F*^2^ > 2σ(*F*^2^)] = 0.063	Hydrogen site location: inferred from neighbouring sites
*wR*(*F*^2^) = 0.171	H-atom parameters constrained
*S* = 1.14	*w* = 1/[σ^2^(*F*_o_^2^) + (0.0839*P*)^2^ + 0.1163*P*] where *P* = (*F*_o_^2^ + 2*F*_c_^2^)/3
2142 reflections	(Δ/σ)_max_ = 0.001
136 parameters	Δρ_max_ = 0.34 e Å^−^^3^
0 restraints	Δρ_min_ = −0.23 e Å^−^^3^

### Special details

**Table:**

Geometry. All e.s.d.'s (except the e.s.d. in the dihedral angle between two l.s. planes) are estimated using the full covariance matrix. The cell e.s.d.'s are taken into account individually in the estimation of e.s.d.'s in distances, angles and torsion angles; correlations between e.s.d.'s in cell parameters are only used when they are defined by crystal symmetry. An approximate (isotropic) treatment of cell e.s.d.'s is used for estimating e.s.d.'s involving l.s. planes.
Refinement. Refinement of *F*^2^ against ALL reflections. The weighted *R*-factor *wR* and goodness of fit *S* are based on *F*^2^, conventional *R*-factors *R* are based on *F*, with *F* set to zero for negative *F*^2^. The threshold expression of *F*^2^ > σ(*F*^2^) is used only for calculating *R*-factors(gt) *etc*. and is not relevant to the choice of reflections for refinement. *R*-factors based on *F*^2^ are statistically about twice as large as those based on *F*, and *R*- factors based on ALL data will be even larger.

### Fractional atomic coordinates and isotropic or equivalent isotropic displacement parameters (Å^2^)

**Table:**

	*x*	*y*	*z*	*U*_iso_*/*U*_eq_	
S1	0.16597 (7)	0.6404 (2)	1.04023 (4)	0.0663 (4)	
O1	0.25185 (19)	0.2798 (6)	0.84661 (10)	0.0637 (7)	
N1	0.14062 (19)	0.3425 (5)	0.92651 (10)	0.0425 (6)	
H1A	0.0767	0.2852	0.9388	0.051*	
N2	0.30760 (19)	0.6294 (5)	0.94925 (11)	0.0432 (6)	
H2A	0.3149	0.5546	0.9112	0.052*	
C1	0.0805 (3)	−0.0367 (8)	0.76267 (15)	0.0643 (10)	
H1	0.1454	0.0344	0.7464	0.077*	
C2	−0.0026 (4)	−0.2077 (10)	0.72171 (17)	0.0836 (13)	
H2	0.0062	−0.2506	0.6779	0.100*	
C3	−0.0988 (3)	−0.3159 (8)	0.74552 (18)	0.0709 (11)	
H3	−0.1557	−0.4286	0.7176	0.085*	
C4	−0.1102 (3)	−0.2566 (8)	0.81058 (16)	0.0563 (8)	
H4	−0.1742	−0.3323	0.8270	0.068*	
C5	−0.0271 (3)	−0.0849 (7)	0.85172 (14)	0.0465 (7)	
H5	−0.0352	−0.0467	0.8958	0.056*	
C6	0.0684 (2)	0.0310 (6)	0.82792 (13)	0.0422 (7)	
C7	0.1604 (2)	0.2252 (7)	0.86697 (13)	0.0425 (7)	
C8	0.2103 (2)	0.5429 (6)	0.97010 (13)	0.0407 (7)	
C9	0.4014 (2)	0.8203 (6)	0.97749 (12)	0.0386 (7)	
C10	0.4932 (2)	0.8417 (7)	0.94239 (13)	0.0478 (8)	
H10	0.4887	0.7333	0.9029	0.057*	
C11	0.4091 (2)	0.9828 (7)	1.03623 (13)	0.0472 (8)	
H11	0.3490	0.9734	1.0610	0.057*	

### Atomic displacement parameters (Å^2^)

**Table:**

	*U*^11^	*U*^22^	*U*^33^	*U*^12^	*U*^13^	*U*^23^
S1	0.0557 (5)	0.0955 (8)	0.0554 (5)	−0.0286 (5)	0.0299 (4)	−0.0292 (5)
O1	0.0538 (13)	0.0916 (19)	0.0525 (12)	−0.0245 (12)	0.0274 (10)	−0.0201 (12)
N1	0.0372 (12)	0.0525 (16)	0.0409 (12)	−0.0114 (11)	0.0155 (10)	−0.0048 (11)
N2	0.0427 (13)	0.0514 (15)	0.0378 (12)	−0.0114 (11)	0.0136 (10)	−0.0052 (10)
C1	0.068 (2)	0.081 (3)	0.0476 (18)	−0.0252 (19)	0.0218 (15)	−0.0127 (17)
C2	0.104 (3)	0.103 (3)	0.0458 (19)	−0.041 (3)	0.0214 (19)	−0.025 (2)
C3	0.071 (2)	0.074 (3)	0.064 (2)	−0.024 (2)	0.0035 (18)	−0.0142 (19)
C4	0.0462 (18)	0.060 (2)	0.064 (2)	−0.0110 (15)	0.0133 (15)	−0.0022 (17)
C5	0.0475 (16)	0.0525 (19)	0.0415 (14)	−0.0021 (14)	0.0131 (12)	−0.0058 (13)
C6	0.0457 (16)	0.0428 (17)	0.0390 (14)	−0.0002 (13)	0.0103 (12)	−0.0013 (13)
C7	0.0452 (16)	0.0465 (18)	0.0373 (15)	−0.0027 (13)	0.0120 (12)	0.0019 (12)
C8	0.0399 (15)	0.0432 (17)	0.0401 (14)	−0.0026 (13)	0.0103 (11)	0.0044 (12)
C9	0.0399 (15)	0.0430 (17)	0.0333 (13)	−0.0032 (12)	0.0078 (11)	0.0037 (12)
C10	0.0487 (17)	0.058 (2)	0.0397 (15)	−0.0101 (14)	0.0149 (13)	−0.0109 (14)
C11	0.0425 (16)	0.060 (2)	0.0433 (15)	−0.0087 (14)	0.0188 (12)	−0.0040 (14)

### Geometric parameters (Å, °)

**Table:**

S1—C8	1.656 (3)	C3—C4	1.373 (5)
O1—C7	1.227 (3)	C3—H3	0.9300
N1—C7	1.374 (3)	C4—C5	1.380 (4)
N1—C8	1.403 (3)	C4—H4	0.9300
N1—H1A	0.8600	C5—C6	1.386 (4)
N2—C8	1.330 (3)	C5—H5	0.9300
N2—C9	1.415 (3)	C6—C7	1.481 (4)
N2—H2A	0.8600	C9—C11	1.385 (4)
C1—C2	1.376 (5)	C9—C10	1.385 (4)
C1—C6	1.386 (4)	C10—C11^i^	1.376 (4)
C1—H1	0.9300	C10—H10	0.9300
C2—C3	1.381 (5)	C11—C10^i^	1.376 (4)
C2—H2	0.9300	C11—H11	0.9300
			
C7—N1—C8	128.9 (2)	C6—C5—H5	119.7
C7—N1—H1A	115.5	C5—C6—C1	118.6 (3)
C8—N1—H1A	115.5	C5—C6—C7	125.0 (2)
C8—N2—C9	132.6 (2)	C1—C6—C7	116.5 (3)
C8—N2—H2A	113.7	O1—C7—N1	120.9 (3)
C9—N2—H2A	113.7	O1—C7—C6	120.8 (2)
C2—C1—C6	120.8 (3)	N1—C7—C6	118.3 (2)
C2—C1—H1	119.6	N2—C8—N1	113.9 (2)
C6—C1—H1	119.6	N2—C8—S1	127.6 (2)
C1—C2—C3	120.1 (3)	N1—C8—S1	118.45 (19)
C1—C2—H2	120.0	C11—C9—C10	118.2 (2)
C3—C2—H2	120.0	C11—C9—N2	126.0 (2)
C4—C3—C2	119.7 (3)	C10—C9—N2	115.8 (2)
C4—C3—H3	120.1	C11^i^—C10—C9	122.5 (3)
C2—C3—H3	120.1	C11^i^—C10—H10	118.8
C3—C4—C5	120.2 (3)	C9—C10—H10	118.8
C3—C4—H4	119.9	C10^i^—C11—C9	119.3 (2)
C5—C4—H4	119.9	C10^i^—C11—H11	120.4
C4—C5—C6	120.6 (3)	C9—C11—H11	120.4
C4—C5—H5	119.7		
			
C6—C1—C2—C3	−0.4 (7)	C5—C6—C7—N1	−11.9 (4)
C1—C2—C3—C4	−1.2 (7)	C1—C6—C7—N1	168.0 (3)
C2—C3—C4—C5	1.2 (6)	C9—N2—C8—N1	−178.7 (3)
C3—C4—C5—C6	0.4 (5)	C9—N2—C8—S1	0.3 (5)
C4—C5—C6—C1	−1.9 (5)	C7—N1—C8—N2	0.7 (4)
C4—C5—C6—C7	177.9 (3)	C7—N1—C8—S1	−178.4 (2)
C2—C1—C6—C5	2.0 (5)	C8—N2—C9—C11	−3.8 (5)
C2—C1—C6—C7	−177.9 (3)	C8—N2—C9—C10	175.8 (3)
C8—N1—C7—O1	3.9 (5)	C11—C9—C10—C11^i^	0.0 (5)
C8—N1—C7—C6	−176.1 (3)	N2—C9—C10—C11^i^	−179.6 (3)
C5—C6—C7—O1	168.1 (3)	C10—C9—C11—C10^i^	0.0 (5)
C1—C6—C7—O1	−12.0 (5)	N2—C9—C11—C10^i^	179.6 (3)

Symmetry codes: (i) −*x*+1, −*y*+2, −*z*+2.

### Hydrogen-bond geometry (Å, °)

**Table:**

*D*—H···*A*	*D*—H	H···*A*	*D*···*A*	*D*—H···*A*
N2—H2A···O1	0.86	1.85	2.590 (3)	144
C11—H11···S1	0.93	2.56	3.215 (3)	128
C5—H5···S1^ii^	0.93	2.84	3.567 (3)	136

Symmetry codes:  (ii) −*x*, −*y*+1, −*z*+2.
